# The development of a regional network for health care research in Hamburg, Germany

**DOI:** 10.1093/eurpub/ckac131.311

**Published:** 2022-10-25

**Authors:** C Lindemann, S Busch, T Bott, A Meusch, B Loewe, M Scherer, O von dem Knesebeck, M Haerter

**Affiliations:** 1 University Medical Center Hamburg-Eppendorf, Centre for Health Care Research, Hamburg, Germany; 2 University of Applied Sciences, Competence Center Gesundheit, Hamburg, Germany; 3 AOK Rheinland/Hamburg, Hamburg, Germany; 4 University Medical Center Hamburg-Eppendorf, Department of Psychosomatic Medicine and Psychotherapy, Hamburg, Germany; 5 University Medical Center Hamburg-Eppendorf, Department of General Practice and Primary Care, Hamburg, Germany; 6 University Medical Center Hamburg-Eppendorf, Institute of Medical Sociology, Hamburg, Germany; 7 University Medical Center Hamburg-Eppendorf, Institute and Outpatients Clinic of Medical Psychology, Hamburg, Germany; 8 TK, Hamburg, Germany

## Abstract

**Background:**

At the University Medical Center Hamburg-Eppendorf (UKE), health care research has been established as one of five research priorities recommended by the Research Council with the founding of the Center for Health Care Research (CHCR) in 2006. The CHCR was involved in numerous research projects with the focus on strengthening regional networking. Despite the numerous initiatives, there is still potential for improvement with regard to a systematic and sustainable exchange in the region of Hamburg, Germany.

**Methods:**

These requirements led to the initiation of the ‘Hamburg Network Health Services Research (HAM-NET)'. The mission of HAM-NET is to build an open forum for all relevant institutions, to concentrate their interests and needs in health services research and to promote and conduct innovative, efficient, needs-based and patient-centred health services research projects in the metropolitan area of Hamburg. Three main tasks were appointed: 1) linking health care research to relevant regional institutions, 2) promoting research activities and new fields of research and 3) using methodological expertise to promote young scientists.

**Results:**

By today 40 institutions from all sectors of health care joined HAM-NET. The regularly general meetings offer exchange and advice. Internal communication is promoted by mailing lists and newsletters. Also, HAM-NET presents itself with a website, logo and by organizing recurring events and participating in international and national congresses and networks. Within two funding phases a total of four overarching research projects were developed and implemented. Furthermore, a person with lived experience committee was established.

**Conclusions:**

For the further development of health care research as one the core disciplines of public health a regional network with an efficient infrastructure is needed. HAM-NET promotes this with the implementation of an innovative, efficient and patient-oriented network.

**Key messages:**

• Regional networks help to integrate multiple public health initiatives and community stakeholders.

• Public health networks can be established on multidisciplinary cooperations in different out- and inpatient sector levels.

